# The indigenous knowledge system of Credo Mutwa: a pedagogical challenge in higher education in South Africa

**DOI:** 10.1007/s41297-023-00182-2

**Published:** 2023-03-28

**Authors:** Tebogo Victoria Kgope

**Affiliations:** University of South Africa, Pretoria, South Africa

**Keywords:** Africanisation, Curriculum, Theory, Praxis

## Abstract

This article discusses the vast indigenous knowledge system of the late Sanusi Credo Vusamazulu Mutwa as a critical and relevant pedagogy that can enhance the transformation agenda of an African university. It argues for the inclusion of Mutwa’s indigenous knowledge to form part of knowledge that will interface with other knowledges and be included in institutions of higher learning. It argues that the constant drives for an African university with an Africanised and decolonised curriculum must be entrenched by recognising indigenous knowledge holders as public intellectuals who can through social cohesion engage with scholars and students in higher education. It is a fact that indigenous wisdom remains a challenge and a contested terrain in higher education as it is observed that not only have institutions of higher learning admitted to the calls to decolonise, Africanise and indigenise the curriculum but these calls have been met with failures to recognise the intellectual emancipating knowledge systems of their very own African intellectuals. It is these tendencies that have led to the blindness of these spaces not to adequately recognise the canonical knowledge of Credo Mutwa, an indigenous knowledge system which innervates through various fields of studies.

## Introduction

It would be proper to start by engaging an African epistemic framework with African thought patterns that use critical social theory, herein the perspective of South Africa. It is pertinent to point out that executing such requires one to fundamentally ask relevant questions. Who are the relevant people that should (re)generate the pedagogical justification that supports nations, national treasures, intellectual heritage traditions, ancient wisdom, laws, the academy at large etc.? Moreover, through the baton of passing knowledge from one generation to another, how should nations and the academy or other innovations realise the manifestation of redressing, restoring, and re-membering indigenous knowledge and wisdom in its focus and emphasis? Keane et al. (2016, p. 164) concur that indigenous knowledge stories as passed from one generation to the other are embedded with conceptions of reality. An ancestor in the name of Biko (1978, p. 26) conscientised people that it must be known that “on the whole, any country in Africa, in which the majority of the people are African must inevitably exhibit African values and be truly African in style”. Similarly, a sagacious indigenous knowledge holder and philosopher such as Credo Vusamazulu Mutwa foregrounded the pan-Africanism oeuvre through African indigenous knowledge systems. It is such privileging statements of African thought patterns, values, customs, lifestyle and overly in the domain of indigenous knowledge that have been subjugated and silenced as aspects of transversal knowledge enterprise. This behoves the academy to ask those who were at the warpaths for us, or those who have been in the trenches for South Africa, and those who are without any doubt the relevant and the chosen ones, how often and when have they preferred things that are African? Mutwa (1964, p. 654) argues that “knowledge is controlled among the african people by the orders of the chosen ones” that is those who are initiated with the distinctive nature of ancient indigenous knowledge.

In his seminal work, renowned Sanusi Credo Vusamazulu Mutwa asks, what was there for the modern African to look back upon? In an engaged scholarship indaba held in 2021 at the University of South Africa, his daughter an indigenous knowledge healer Makhosi Vulamasango Mutwa defined the term Sanusi as someone who could smell things from afar. Makanya (2020) alluded to the term to represent “one who lifts us and a healer who reveals that which is hidden, such as mysteries erased by history, that is, someone who tells us about the future”. Mutwa asked “if the french and russian revolution or the industrial revolution and the american war of independence have little significance to the intellectual heritage of an african, what would be meaningful and relevant to someone who has an african ancestry” (Mutwa, 1964, p. 690). Advancing these ventilating remarks from Mutwa indicates that there should be a palpable African episteme that projects a balance between the prevalence of dominant knowledge opposed to how African ways of knowing are discharged. Can such relevance of African episteme be found in the archives of the academy? In stating for support of indigenous knowledge, Joseph (2018) asserts that:

Black students deserve a curriculum that critically “re-members” their African heritage knowledge, connects them with accurate information that enriches their scholarship and meaningfully identifies their connection to the African Diaspora while deepening their sense of self-worth and efficacy.

An emancipatory pedagogy includes the marginalised knowledge from the locality, and the Global South that the dismembered would have a nexus with their social and ontological being. Chilisa & Mertens (2021, p. 247) argue that to attain credibility in centring indigenous knowledge, evaluation mechanisms that encourage the use of people’s living experiences, and indigenous knowledge will reveal methodologies through which the abstractions of reality can be known. The evaluator epistemic branches they list include social accountability as a root source; the methods division; the use division, needs and context division, values, and social justice division (Chilisa & Mertens, 2021, p. 245). Similarly in *Indaba, My Children* (1964), a seminal book by Mutwa a healer of the highest order is specifically meant for African people not to forget their own ways of knowing and living, it is a compendium of indigenous stories and experiences relating to indigenous folklore and philosophies.

Scholarly traditions within disciplinarity, interdisciplinarity, multi-disciplinarity and trans-disciplinarity are challenged to inculcate African perspectives in their curriculums. Although Nabudere (2011, p. 90) posits that “transdisciplinarity is just one of the mechanisms of fighting the rigidity of the modern ‘scientific method’ that focuses narrowly on the ‘disciplines’ that were [colonially] ‘constructed’ to produce knowledge”. It suggests that the indigeneity of knowledge and its people are what should be traced to advance and produce knowledge. Not only is this a paradigm shift across disciplines alongside the calling as a gift, but indigenous knowledge systems deeply rooted in indigenous wisdom and culture remain to be contested. Their contestations have permitted the ignorance and lack of acknowledgement in venerating saints, seers and sages such as Credo Mutwa. When Mutwa (1964, p. 690) questions if Africans can find the knowledge that will concern and remain meaningful for them in a library, or in the archives of our academic institutions in accessing indigenous knowledge systems he was on point. Mutwa was ahead of his time. In terms of honorary accolades, it becomes fundamentally critical to question why a country such as South Africa has not bestowed Mutwa, a rare Sanusi and indigenous knowledge practitioner with the highest national order of providence. It is a matter of principle on how a country emerges from where it has been, under the infamous dark cloud of colonialism, apartheid and towards a new dispensation that was termed democracy. Also, the often-quoted notion of seeking African solutions for African problems has yielded less impact and benefits given the ongoing existential African realities that depict a country that remains unequal in various aspects.

Mutwa is not only hailed as an African indigenous healer but more uniquely as a Sanusi healer; the accolade of a Sanusi also describes the highest order and calling of an indigenous knowledge healer and holder and Mutwa was one such figure. Amongst the teachings that Mutwa has taught the nation and the world over was the significance of peace. Peace can here be expanded as being absent in terms of advancing knowledge production in indigenous knowledge due to contestations that are rife in this crusade. However, it must be mentioned that since his knowledge is concerned, it cannot be hierarchised or compared or even subjected to questioning its validity for that matter. But then again, how does a nation reach a plateau of a peace treaty amid heavy clouds of violence and polarities. In many predictions, prophecies and visions Mutwa had, violence as a current aggravating matter affecting all societies was highly praised. In Zulu Shaman, Mutwa (2003) writes on violence in the South African landscape that for this violence to end there needs to be unity amongst different cultures and races. In other words, the question of African indigenous healing is very crucial to be grasped at the level of Mutwa’s body of knowledge. This is such because post-1994 South Africa emerged with the recognition of African indigenous healing systems and the national recognition of the indigenous knowledge systems (IKS) policy. The indigenous knowledge systems policy enacted by the Department of Science Technology (2004) gives credence to interfacing indigenous knowledge systems with other knowledge systems including the participation of indigenous knowledge systems practitioners and holders on various platforms. It was the African National Congress (ANC) that privileged talks on the incorporation and integration of African traditional healing systems into the national healthcare programmes (Tjale & De Villiers, 2004). The new dispensation also experienced and continues to experience a proliferation of African traditional healing. This new wave of continual proliferation brought with it a radar or surveillance that would question the authenticity of African indigenous knowledge systems and their various institutional bodies. Underpinning these background cues are taken from Mutwa’s wisdom in indigenous knowledge healing, cosmogony, mythology and other ways of deep knowledge. Furthermore, what needs to be talked to as a guideline or framework for African indigenous healing epistemologies is the recognition of the highest calling within the guilds of African indigenous knowledge systems. And through the lens of the Sanusi as a healer, prophet, seer, sage, nations and country’s embodiment of seeking deep hidden knowledge and wisdom.

## Background

Mutwa established a liberating pedagogy for all humanity; hence, this article promotes his vast indigenous knowledge as a critical and relevant pedagogy in an institution of higher learning. It argues for the insertion of his complex and vast indigenous knowledge to form part of critical pedagogy that can be incorporated in higher institutions of learning. The article also argues that the drives for an African university with an Africanised or decolonised curriculum must be entrenched in acknowledging indigenous knowledge holders as public intellectuals who can engage with scholars in an academic space. The indigenous knowledge guild of Mutwa is an engaged scholarship project conducted at the University of South Africa (UNISA) in the form of conversational indabas or seminars. It is envisaged that the project will contribute to the vast indigenous knowledge canon of Mutwa in the academy to interface his indigenous knowledge with the formal knowledge of the academy for sustainable use.

Regarding contested knowledge systems such as the domain of African indigenous epistemologies with research into this field that is often frowned upon, it is observed that not only have institutions of higher learning admitted to the calls to decolonise, Africanise and or indigenise the curriculum, but these calls have been met with limited calls to recognise the intellectual emancipatory knowledge crafts of their very own African intellectuals. On the other hand, this paralysis takes place while higher institutions of learning continually debate and talk about inculcating modes of African thought patterns in the academic discourse. The frequency at which these discourses are entertained has a periodicity of taking place in modes of what I would call window-dressing mannerisms. For example, addressing these social injustices only when it is heritage month or when the calendar highlights the struggle period of the past, often with the exclusion of mentioning pillars of what would entail an African framework as a liberating pedagogy. Also, what remains problematic is the constant mentioning of notions such as “African problems need African solutions” a notion that is also merely used as a window dressing maxim, while on the sideways, Africans paralytically look to the west for African solutions, indaba. At the core of the promotion of Mutwa’s indigenous knowledge guild is the curriculum transformation that is positioned to align an African framework and pedagogy that locates existential African realities.

It is fundamental to clarify that the current academy or institutions of higher learning present themselves as spaces of think tanks that produce knowledge and spaces that produce alumni that end up in the periphery and zone of unemployment. Unemployment often arises because of disjunctures between African realities and the colonially constructed disciplines. This statement challenges higher institutions of learning to rethink and trace what has been the actual impact on educational outcomes to date.

## Problem statement

The indigenous knowledge guild of Credo Mutwa as a pedagogical challenge to institutions of higher learning is an umbrella term for all knowledge systems that bear the nucleus of indigeneity as a knowledge base. The article emerges as a result of tendencies from institutions of higher learning with constant utilisation of terms such as an idea of an “african university; africanisation and or decolonisation” (Mazrui, 2003; Ndlovu-Gatsheni, 2017, pp. 56, 61, 67; Thaver, 2019, p. 117) but with the very limited or small scale of an African indigenous knowledge base that identifies a framework of reference and liberation. These tendencies have led to the blindness of these spaces not to recognise or honour the knowledge canon of Credo Mutwa, an indigenous knowledge guild which innervates through various fields of studies. The continual paralysis and poverty in inculcating indigenous African knowledge systems demonstrate and indicate that the redress for the injustices of the past still depicts imbalances towards the indigenous knowledge guild of the renowned sage and ancient knowledge keeper. Mazrui (2003, p. 157) has in the past also emphasised that since “modernity is here to stay, the task is to decolonize it and be safe from an excessive eurocentric world culture”. Similarly, Mkandawire (2011, p. 20) attested that “it should be clear that Africa’s self-understanding and its legibility to others will need a large dose of local scholarship”. It is in this vein that Mutwa’s indigenous knowledge system should form part of enhancing African scholarship in its various localities. The Mutwa(ian) indigenous knowledge guild is immense and complex but it is not without mechanisms that can enable transformation in the academy.

Problematising this article from the College of Graduate Studies School of Interdisciplinary Research and Graduate Studies at the University of South Africa in adherence to the transformation agenda of the institution is that few dependable academics work in the domain of African indigenous epistemologies and indigenous knowledge research. This was not only a misnomer for an institution that calls itself an African university, but it is these postures that lead to failure in transforming the academy. Also, one of the challenges in disseminating indigenous African knowledge and epistemologies was identified as the failure of academics to honour invitations to present at seminars.

Therefore, this article provides credibility to the indigenous knowledge guild of Credo Mutwa, whose passing received minute recognition from higher institutions of learning despite the neglect of his knowledge base in these spaces. Through indabas or seminars in the form of conversational virtual platforms, the indigenous knowledge guild of Mutwa engaged and formed social cohesion relations between the academy and the public intellectuals as indigenous knowledge holders and well-informed experts in the terrain of African epistemologies.

## Objectives

This article aims at developing and promoting a relationship between the public intellectual and a scholar (Mamdani, 2016). The concept of the public intellectual and the scholar is derived from the Mamdanian maxim of practising modes of “theorising our reality” through enhancing the emancipative indigenous knowledge of Mutwa. Mamdani (2016, p. 81) confirms that certainly “in a colonial context, the tension between the scholar and the public intellectual reflected a wider divide, between one who produces theory and one who applies it”. The engaged scholarship in promoting Mutwa’s indigenous knowledge regards Mutwa as a public intellectual in what Mamdani (2016) attributes as “a social critic, the producer of knowledge, and of decolonized subjects who are either public intellectuals and or scholars”. Similarly, Reddy (2021) also states that public intellectuals have on a broader scale imparted thought patterns that have changed South African society in a positive shape, irrespective of “whether this is about how we treat one another, how we manage the great challenges of our time or showing us how music and art can help us navigate ideas that can change the world”. A public intellectual is also equivalent to someone whose purpose represents the people and issues that are frowned upon, suppressed and swept under the carpet.

The need to redress the injustices of the past is to articulate and promote silenced and marginalised knowledge systems of the Global South. Boaventura de Sousa Santos (2007, 2016) asserts that the Global South is comprised of marginalised knowledge systems of the voiceless, the silenced and the massacred. Masoga (2002) refers that marginalised knowledge, herein, African epistemologies are a contested terrain with failure of recognising indigenous knowledge and African intellectuals. Dei (2012) and Emeagwali and Dei (2014) also assert that the promotion and development of indigenous knowledge research and African epistemologies must be transcended by those who are the knowledge holders of this domain of knowing.

## Literature survey

According to Dei (2012; Emeagwali & Dei, 2014), scholars in the African academy have recognised that the promotion and development of indigenous knowledge research and African epistemologies must be transcended by those who are the knowledge holders of this domain of knowing. However, Masoga (2002) has alluded to the nature of this domain of knowledge to be often side-lined. Again, to coincide with Nabudere’s conceptual framework of using Africans’ lived historical situation as an emancipatory epistemology, it is (Ndebele in Manganyi, 2019, p. 119) who affirms that it would appear that what constituted the need to transform has a bearing on relieving “black pain” which was constitutionally a historical question. The remnants of these historical questions and conditions of African people with their indigenous epistemologies are embraced by Mamdani (2016, 2019) on asserting for an African university to close the gap between the public intellectual and the scholar as they both must learn to theorise and re-write their everyday realities. This Mamdanian maxim is also purported to be an emancipatory mode of transcending marginalised epistemologies such as African indigenous epistemologies. While modes and notions to transform or Africanise knowledge and the academy may seem to be pronounced in the current timelines, in his seminal work Biko (1978, p. 72) advocated in *I Write What I Like* that the spirit of African liberalism must always prevail. It can be learned that from these propositions even Manganyi (2019, pp. 40–41) once lamented that the quest of Africans in South Africa was reduced by Africans themselves abandoning their African traditional approaches at the community level. Manganyi (2019, p. 42) gives an account where this posture that dismembers the Africans from African values and modes of knowing leads to a disconnection of being in the world. Although Manganyi (2019) and others may illuminate where the problems arise, this is often from social constructs of the world the Africans were socialised into. However, Ndlovu (2018) in acknowledging these social constructs due to the Eurocentric epistemology argues that the ideas of Africanising or decolonising may not be adequate if the dominant knowledge is not transcended. By this Ndlovu (2018, p. 96) implies that failure to beat the dominant western-centric knowledge while advocating for African epistemologies and indigenisation of the curriculum will only result in the quagmire of “repetition without change”. To advance this argument would indicate that if Africans are not aware of how Eurocentric knowledge distorted their African modes of knowing, the repetition and subscription to western-centric thought patterns will continue without fail. It is herein that Ndlovu (2018, p. 110), Ndlovu-Gatsheni (2017) and others like Mignolo (2009) and Gordon (2014) propose for a process to unlearn what has been the subjection of colonising knowledge and to re-learn from epistemologies that have been marginalised.

Furthermore, it is a fact that the subject matter of Credo Mutwa’s indigenous knowledge guild should be observed as an emancipatory African epistemology that privileges African thought patterns and modalities to relearn and reclaim African values and modes of knowing in their various terrains. In their veracity, notions of Africanising, decolonising and using African epistemologies are aimed at seeking the truth. And people with positive aspects in their minds are also according to Mutwa “those who seek for the truth, their hearts are one and clear” (Podolecka, 2018, p. 151).

## A defence of Mutwa(ian) pedagogy of emancipation

As a pedagogy of liberation, Mutwa(ian) indigenous knowledge guild and pedagogy is immense and diverse and can serve as an instrument that develops the indigenisation of various disciplines and curriculum reform. As Marwala (2021, p. 53) asserts on the propositions of names that carry “the suffix ‘nium’ in science and technology that it usually means the prefix is the important component”. Therefore, this statement would indicate that adding the suffix “ian” positions and coins a domain of knowledge that is derived from Mutwa himself. Mutwaian indigenous knowledge guild asks us:

why would africans allow anyone, however powerful, to distort history in any way because they strongly believe that by doing so they attempt to deceive the gods with the result that the Spirits of the Tribe would be highly offended? (Mutwa, 1964, p. 216).

In other words, this article argues why institutions of higher learning speak of transformation but offend by exclusion the ontological corpus of the indigenous knowledge of Mutwa in setting an African epistemic framework as critical pedagogy. Ramose (2002, p. 1) argues that to achieve a state of emancipatory freedom “one of the requirements is that africans should take the opportunity to speak for and about themselves and in that way construct an authentic and truly african discourse about Africa”. Ramose (2002) also sanctions us to recognise that having the potential and “quality to reason” defends all efforts of transformation and gives impetus to canonical works of Mutwa’s epistemology and ontology. What is the community knowledge of this guild that can be interfaced with formal knowledge with an intent to contribute to a curriculum transformation agenda of an African university? The answer lies in what differentiates Mutwa’s indigenous knowledge system from other ancient modes of knowledge.

## Theoretical and methodological underpinnings

An emancipatory knowledge research paradigm is not only often subscribed to terms such as being confined as a qualitative approach (Ledwith, 2017; Gunbayi & Sorm, 2018), in this article the research paradigm in applying and recognising the indigenous knowledge guild of Mutwa is of a (post)metaphysical and philosophical mode of enriching African epistemologies in higher institutions of learning. Therefore, the emancipatory research paradigm in this mode is appropriate for this engagement since it is a liberating and transcending domain of knowledge.

The mode of engaging in community engagement indabas or conversations as attempts to articulate and promote Mutwa(ian) indigenous knowledge pedagogy is also attributed by Le Grange (2018). According to Le Grange (2018, p. 6) “scholars in South Africa should commit to the complicated conversations that are essential if knowledge advancement in curriculum reform and studies is to be developed”. It is through conversational indabas that knowledge is studied and shared by public intellectuals to advance modes of operations that will be considered to affect the utilisation and application of knowledge that has been marginalised. As Masoga (2002) aptly affirms:

researchers in the field of Indigenous Knowledge Systems should be reminded to tread carefully in the area of epistemology. They have to form a ‘rapport’ of ‘trust’ in engaging ‘local research participants’ because it is the latter’s space; they own and direct it. Who can claim their space? Surely, only they themselves! They (local research participants) have the ability to direct their margin space to the centre.

Mutwa concurs that (1964, p. 657) the Africans conduct research in a different style as compared to Eurocentric ways of knowing. His conceptual apparatus argues for a pedagogy of liberation.

Le Grange (2018, p. 7) emphasises that:

Frank and ongoing self-criticism is an important dimension of complicated conversations because it mitigates hierarchical power relations that could impede productive conversations from happening. Power relations are always present when humans engage in educational exchanges. However, complicated conversations are constructed to lessen hierarchical power relations and their colonising effects.

What Le Grange (2018) is advocating for here is analogous to the mode of positioning criticism through modes of writing back to the self, particularly when the self represents the academy, scholars and students. This mode of writing would entail voicing the discomforts or humiliations that lead to the inadequate address of indigenous knowledge system as compared to western-centric paradigms. Although in Le Grange’s emphasis, self-criticism is at the level that is similar to conversational indabas or interviews that engages both the scholar and the public intellectual. Fanon writes about the necessities of African literature or philosopher having an encounter with some Platonian theorist or works (Fanon, 1952, p. 180). Therefore, through the question that Mutwa posed to African indigenous people about why they would tolerate the hegemony of western-centric paradigms to misrepresent their history, a critical response by Fanon (1952) emphasises that these conversational indabas are imperative so that there could be an atmosphere where Mutwa(ian) pedagogy of liberation sails on the same shores with canonical scholars of Eurocentric worldviews.

To commensurate the urgency in privileging African indigenous epistemologies, the following sections succinctly engage what Mutwa foretold and understood as visions, mysteries and metaphysical philosophies of knowing. The topics identify with the critical social theory of South Africa and the global world on everyday existential and ontological realities. The aim is to purposively demonstrate that Mutwa is a rare Sanusi that should not be mocked or scoffed at, as he said elsewhere about African history.

## Decolonisation of the mind and education

Mutwa has extensively weighed in on how Eurocentric knowledge systems have negated and permitted Africans to despise their ways of knowing. Mutwa (2003, p. 31) was of the idea that “this is why we never realised the tremendous store of knowledge that lies hidden in the minds of those people who are called sangomas and traditional healers”. In respect of the knowledge regarding healing, knowing oneself as a people, as a nation and as a country Mutwa contextually and conceptually conferred South Africa and Africa with a conceptual framework that aligns with how we can truly be ourselves, and what the core of humanity is meant to be. He has written broadly in *Indaba, My Children* (1964) on what theories can be applied instead of the Eurocentric paradigms that are opposed to finding solutions to African problems. In the book, again ahead of his time, he wrote about the decolonisation of education that the book was to debunk the distorted, skewed curricula that were taught in schools and churches to African people (Mutwa, 1964, p. 533). Also, in 2015 South African universities demonstrated through the #RhodesmustFall and fees must fall campaigns the disjuncture of what is taught in line with African realities (Mavunga, 2019). These were the student movements which students used to set the tone in criticising the state of higher learning institutions as colonial while protesting for a more decolonial education that speaks to their everyday existential and ontological realities. The theories are what social policy should engage for ease of implementation which is what is lacking in many various policies that are adopted.

## We have not grasped the core of *Indaba, My Children*: unpacking Credo Mutwa a Sanusi

Amongst all the books he authored, Mutwa’s book *Indaba, My Children* (1964) is poised more like an acapella with a collection of meaningful indigenous African knowledge systems. This is such because the book does not mince its words, one will grasp the words and the meaning of the knowledge the words convey. Therefore, like an acapella song which makes one appreciate the voice of the singer, his ancestral voices coming as stories, predictions, warnings and the vast knowledge that innervates all spheres of life were not adequately valued or appreciated. The indigenous stories as worthy as they are, remain to be a complex application for consideration in what is meant in the often-quoted notion that African problems need African solutions.

The question is what are we so modestly afraid of? Knowledge systems in his book push us to upgrade indigenous African knowledge systems, the institutions of indigenous healing, indigenous African jurisprudence, indigenous languages, African (religion) or belief systems, African theories and the maintenance of burial grounds which brings up matters of the unresolved land question. While with any upgrade comes a downgrade, in his own acapella words he wrote that “many of the books written by europeans about africans should be relegated to the dustbin” (Mutwa, 1964, p. xviii), and yet the source of knowledge applied for Africans and African problems remains glaringly western in shape, size and structure.

History is factually painful but what are the lessons that can be learned from indigenous African historical foundations? Mutwa said “history cannot be mocked; history is not something that should be scoffed at”, here he was alluding to the imperative need for people not to forget who they are. However, his words fell on deaf ears as what he said was not grasped and valued to the core. For history, as it directs us to the past, it also invokes us to be aware that “that” past is still with us in our everyday realities. This article also serves as a response to defend his liberating legacy which has been mired by pessimists who regarded him as an apartheid apologist. These conjectures alongside what Mutwa throughout has uttered are decisively unpacked and argued in defence of demystifying what came up as an epistemic violation of his knowledge by critics.

We begin by unpacking the utterances that pertain to apartheid and its legacies in explicit terms that speak to our everyday realities, and we end by unpacking the understanding and aligning the defence tools in a manner that is befitting to one who is called a Sanusi to be understood when they communicate. Communication in this sphere is not a casual conversation but an interconnection and a communion with everything (Nabudere, 2011, p. 126).

As a navigator, he socialised *umntu, motho* and Bantu, or people as people who should practise and apply the knowledge of their cultures. His vast knowledge innervates almost all spheres of life and disciplines. It then becomes strange when a Sanusi navigates through deep hidden knowledge that even those in the constructed disciplines due to being fragmented from the core of *Indaba, My Children* (1964) fail to privilege and associate themselves with indigenous knowledge systems for its ascendency. It is this knowledge he offered that not only is liberating but more unmistakably challenging what glaringly are Africa’s everyday realities. It is not adequately convincing that his pedagogy is understood particularly if failures in the past were to introduce his knowledge which directs one to measure the immeasurable. Unpacking what he meant by “mocking and scoffing at history” would be not having learnt the lessons from stories that some of our parents told us when they had to go through the pass-law system during the apartheid years. The current timelines of the global pandemic of COVID-19 reminded some about the pass-law systems, when African people had to negotiate their way, and freedom to open their spaza shops to obtain a pass that comes as a permit.

History was certainly mocked and scoffed at when Mutwa indicated that “evil was upon Black South Africans” as he foretold that Black people will kill one another and that in South Africa when you speak the truth, you are a target to be killed. He further said, “my heart is too angry, I don’t want to talk anymore, it doesn’t help. Some people take me for a joke. If I were to open my chest the country would be shocked”. Again, the ancient wisdom was not appreciated, as it went to deaf ears and eyes. Eyes that were blind that will not see and continue to not see. Using Mutwa’s words “if I were to open my chest the country would be shocked” as much as this was referring to indicating what he sees, the words also initiate us to think of the chest as housing the lungs which offer us the basis to breathe freely, when these organs are compromised not only is life threatened, one suffocates, those who are healers when silenced or muted they suffer more. The current global pandemic chose the respiratory system as a reminder to the “chest of the Sanusi” which in 2013 was closed so that as a country we would not be shocked by what the Sanusi was predicting or warning against. The global community went into shock when a pandemic with the affinity to inflict the lungs struck. It is customary in the domain of indigenous healing to learn from those who are in the realm of the Sanusi(ship) to translate the pandemics’ timeline by alluding that *gona le moya o maswe o fokang*, i.e. there is an evil spirit blowing. Knowing that indigenous knowledge healers also use and depend on the respiratory cavity for the flow of energy and hidden knowledge they transfer. The question is were those in the Sanusi(ship) given a stage to guide leadership in this country? Of what use is knowledge from the institution of indigenous knowledge healing when indigenous knowledge holders as public intellectuals are placed at backstage?

Mutwa also epitomised *taola* or *ditaola* (divination bones). As *taola* and at times talking in the plurality of *ditaola* he examined and diagnosed humanity, and the universe, sharing with humanity *dipheko* (medicines) in the form of words and statements he articulated in various books. He institutionalised the indigenous healing sector as powerful and a science in its own right. As a divining bone, he aimed to observe an atmosphere of peace and humanness for all humanity. As many of us only saw his physicality, however, as one who was imbued with the wisdom of being a Sanusi, as *taola* that sees and reveals the truth he would also characterise all genders in appearance. Symbols of a man, woman, old man or old woman, the transgender group would interchangeably be observed in his dress code, appearance, and how those who guided him would want him like a divining bone to project himself.

## Burial rites rightful for a Sanusi

Mutwa descended to his ancestors on 25 March 2020. The healer and highest Sanusi of a unique kind had openly said he had already broken his oath in writing *Indaba, My Children* (1964) to his nation. He signalled that the indigenous African ways of knowing and doing things should be out there in the open to position indigenous knowledge systems as theories of everyday ordinary practices.

To differentiate burial rites according to people’s culture, it is from burial grounds knowledge that Mutwa taught us that burial grounds are equally, not places to be mocked or scoffed at. Here, he highlights the significance of what remains as the unresolved land question. He problematised this perennial concern by indicating that people cannot be taken away from their land, especially away from the burial grounds of their descendants, without taking proper care of addressing rituals that must be followed. Mutwa warned that such people might as well kill themselves (Mutwa, 1964, p. 580). It may then be interrogated as to who is more likely to lose life tragically and succumb to killing and prevail in hurting other human beings. The burial ground and the land were here being focused on by the Sanusi as everything.

## Will detractors grasp the core of *Indaba, My Children*? A defence for indigenous knowledge systems

It is not for the scholar or public intellectual to question everything and all that Mutwa said. It is social cohesion mechanisms between the scholar and the public intellectual that should expand and interface Mutwa’s indigenous knowledge guild with the formal knowledge systems existing in higher education. As stated by the chairperson of the Credo Mutwa Foundation, Rutendo Ngara at a virtual indaba held at the College of Graduate Studies (2021) themed “emancipating ourselves from mental enslavement: what Mutwa said”, Ngara said Mutwa’s pedagogy “is a pedagogy of the outcast, the misinterpreted, and that his Indigenous Knowledge guild is of a mystery university, and faculties that reside in the spirit of each and everyone one of us (not disciplines)”. She further highlighted that Mutwa’s indigenous knowledge system is initiatory and informs that the future can be innovated and changed in liberating modalities. This implies that Mutwa’s indigenous knowledge system encourages us to rethink, unlearn and relearn what has been subjugated and frowned upon as marginalised knowledge.

During the COVID-19 pandemic, Mutwa is reimagined asking about *abantu bethu bazohlawulwa ngani? batho ba rona batla rufiwa ka eng?* (What is it that will be compensated for our people?) for this sufferance? This and many other scenarios exemplify the forces Mutwa warned about. The question is where are the voices of indigenous knowledge leaders and indigenous healers, and how they have been silenced?

The month in which the Sanusi passed away, 25 March 2020, aged 99, is highly significant in the struggle calendar given the fact that March is dedicated to human rights and social justice. His departure was a gift that solidifies that indeed his life and knowledge base is a path that gave us a framework for human liberation with human rights lores. Crucial to his departure is a call for action not to defy his legacy by mocking and scoffing at his emancipatory pedagogy. It was during the week the nationwide lockdown was announced that Mutwa decided he was drained, that South Africa can go to hell for all he cared because when he spoke he was stoned, he begged to speak and he would be called a wizard.

While the road to redressing the injustices of the past is part and parcel of acknowledging our elders and ancestors, Mutwa was ahead of his time, not only did he disseminate his knowledge through books and digital conversations we have of him. Mutwa gave us a map that we can use to travel through as we seek solutions to our African problems and problems that affect humanity. And as part of a crusade to bring about social justice the journey to travel through this terrain has already begun in 2021 with representatives of the Credo Mutwa Reference Group formerly chaired by South African poet laureate, Mongane Wally Serote, Adv. Sipho Mantula, the Credo Mutwa Foundation and Family. The initiatives are aimed to close the gap that exists between public intellectuals, academics and students in enhancing the emancipatory pedagogy advocated by Credo Vusamazulu Mutwa.

## Conclusion

Put in a forceful mannerism, institutions of higher learning ought not to promote the domain of indigenous knowledge as a matter of ticking the box. The capacity to enhance indigenous knowledge should also be in tandem with writing back to ourselves, particularly those who should be rallying at the forefront in canvassing for indigenous knowledge systems. Writing back to ourselves is synonymous with scholarly work that also positions a critique to African scholars on the developments of capacitating for indigenous knowledge. How will history evaluate us on how this domain of knowledge has been capacitated and applied? In his own words, while defending what should triumph as a subject of elevation, Mutwa said “…see again upon the timeless plain, the massed *impi* of so long ago, so honour always thy ancestral line, and traditions of thy land of birth” (Mutwa, 1964, p. 4). Impi, a Zulu term for war, is at this point referring to the war in decolonising knowledge and colonial modernity. The war in modes of re-membering the ancient wisdom entrenched in the diminishing sphere of indigenous knowledge systems. In many epistemological ways, Mutwa’s emancipatory pedagogies are a call for African indigenous knowledge systems to be re-membered and enacted in higher institutions of learning. It calls for reimaginations of new forms of knowledge that enable the innovations of the African Disneyland and films that can be directed out of his works as with the theatre play of uNosilimela. Kavanagh (2019, p. 7) conveys that Mutwa’s pedagogy of uNosilimela demonstrates that “it is in African forms and content, or storytelling and ritualistic performances that answers will be discovered”.
